# Sublayer-Enhanced Growth of Highly Ordered Mn_5_Ge_3_ Thin Film on Si(111)

**DOI:** 10.3390/nano12244365

**Published:** 2022-12-07

**Authors:** Ivan Yakovlev, Ivan Tarasov, Anna Lukyanenko, Mikhail Rautskii, Leonid Solovyov, Alexander Sukhachev, Mikhail Volochaev, Dmitriy Efimov, Aleksandr Goikhman, Ilya Bondarev, Sergey Varnakov, Sergei Ovchinnikov, Nikita Volkov, Anton Tarasov

**Affiliations:** 1 Kirensky Institute of Physics, Federal Research Center KSC SB RAS, 660036 Krasnoyarsk, Russia; 2 Institute of Engineering Physics and Radio Electronics, Siberian Federal University, 660041 Krasnoyarsk, Russia; 3 Institute of Chemistry and Chemical Technology, Federal Research Center KSC SB RAS, 660036 Krasnoyarsk, Russia; 4 Krasnoyarsk Scientific Center, Siberian Branch, Russian Academy of Sciences, 660036 Krasnoyarsk, Russia; 5 REC «Functional Nanomaterials», Immanuel Kant Baltic Federal University, 236016 Kaliningrad, Russia

**Keywords:** manganese germanide, thin film, MBE, ferromagnetism, sublayer

## Abstract

Mn_5_Ge_3_ epitaxial thin films previously grown mainly on Ge substrate have been synthesized on Si(111) using the co-deposition of Mn and Ge at a temperature of 390 °C. RMS roughness decreases by almost a factor of two in the transition from a completely polycrystalline to a highly ordered growth mode. This mode has been stabilized by changing the ratio of the Mn and Ge evaporation rate from the stoichiometric in the buffer layer. Highly ordered Mn_5_Ge_3_ film has two azimuthal crystallite orientations, namely Mn_5_Ge_3_ (001) [1-10] and Mn_5_Ge_3_ (001) [010] matching Si(111)[-110]. Lattice parameters derived *a* (7.112(1) Å) and *c* (5.027(1) Å) are close to the bulk values. Considering all structural data, we proposed a double buffer layer model suggesting that all layers have identical crystal structure with P6₃/mcm symmetry similar to Mn_5_Ge_3_, but orientation and level of Si concentration are different, which eliminates 8% lattice mismatch between Si and Mn_5_Ge_3_ film. Mn_5_Ge_3_ film on Si(111) demonstrates no difference in magnetic properties compared to other reported films. T_C_ is about 300 K, which implies no significant excess of Mn or Si doping. It means that the buffer layer not only serves as a platform for the growth of the relaxed Mn_5_Ge_3_ film, but is also a good diffusion barrier.

## 1. Introduction

Ferromagnetic manganese germanide Mn_5_Ge_3_ thin films have been the object of close study for about 20 years [1,2], remaining a promising material for spintronic devices [3,4] since they possess the necessary properties [5]. The Curie temperature T_C_, which is 296 K for the stoichiometric composition of a bulk crystal [6], can be increased by doping with iron [7], carbon [8], or other dopants [9]. Furthermore, the magnetic properties of such films can be changed via epitaxial stresses [10]. The simplest method for obtaining Mn_5_Ge_3_ thin films is solid-phase epitaxy, in which germanium and manganese films are annealed on a substrate [10,11,12]. There are also several works on the synthesis of epitaxial films using the molecular-beam epitaxy (MBE) method on semiconductor Ge substrates [13,14,15,16], and even on GaAs(111) and GaSb(001) [17,18]. However, the successful synthesis of Mn_5_Ge_3_ films on Si substrates through direct MBE growth has not been reported, which is most likely due to a rather large lattice mismatch (8%). The solution to this problem will contribute to the creation and study of Mn_5_Ge_3_-based magnetic and spintronic devices compatible with silicon CMOS technology. In our work, we demonstrate the successful synthesis of a highly ordered Mn_5_Ge_3_ film on a Si(111) substrate using the MBE method, which was implemented by finely tuning the buffer layer.

## 2. Experimental Details

Mn_5_Ge_3_ films were synthesized on p-Si(111) substrate using MBE (base vacuum 6.5 × 10^−8^ Pa). Si surface was annealed in a vacuum chamber until the 7 × 7 reconstruction appeared, providing an atomically smooth and clean surface. Then, the silicon substrate was cooled to a temperature of 390 °C, which was kept constant during all experiments. Mn and Ge were co-deposited in certain ratios from Knudsen effusion cells. The process was monitored in situ using reflection high-energy electron diffraction (RHEED). After deposition, the samples were evacuated from the vacuum chamber and thoroughly characterized. X-ray diffraction (XRD) analysis was performed using a PANalytical (Panalytical, Almelo, Netherlands) X’Pert PRO diffractometer equipped with a solid-state detector PIXcel on Cu Ka radiation. Morphology and microstructure of Mn_5_Ge_3_ films were studied with atomic force microscopy (AFM) and transmission electron microscopy (TEM) with help of a NanoInk (DPN 5000 device, NanoInk, Skokie, IL, USA) DPN 5000 instrument and Hitachi (Hitachi, Tokyo, Japan) HT-7700 microscope, respectively. To investigate details of the chemical composition of the thin film obtained, Rutherford backscattering spectroscopy (RBS) was used with helium ions, He+, at 1.504 MeV and a scattering angle of 160° relative to the beam’s propagation direction. The magnetic properties of the prepared films were examined with the help of a vibrating sample magnetometer (VSM) LakeShore (Lake Shore Cryotronics, Westerville, OH, USA) VSM 8600.

## 3. Results and Discussion

**Figure 1:**
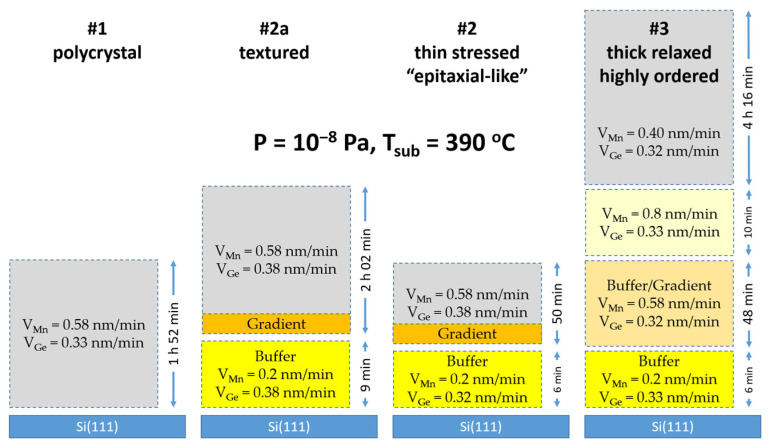
Diagram of process flow for four different samples.

**Figure 2:**
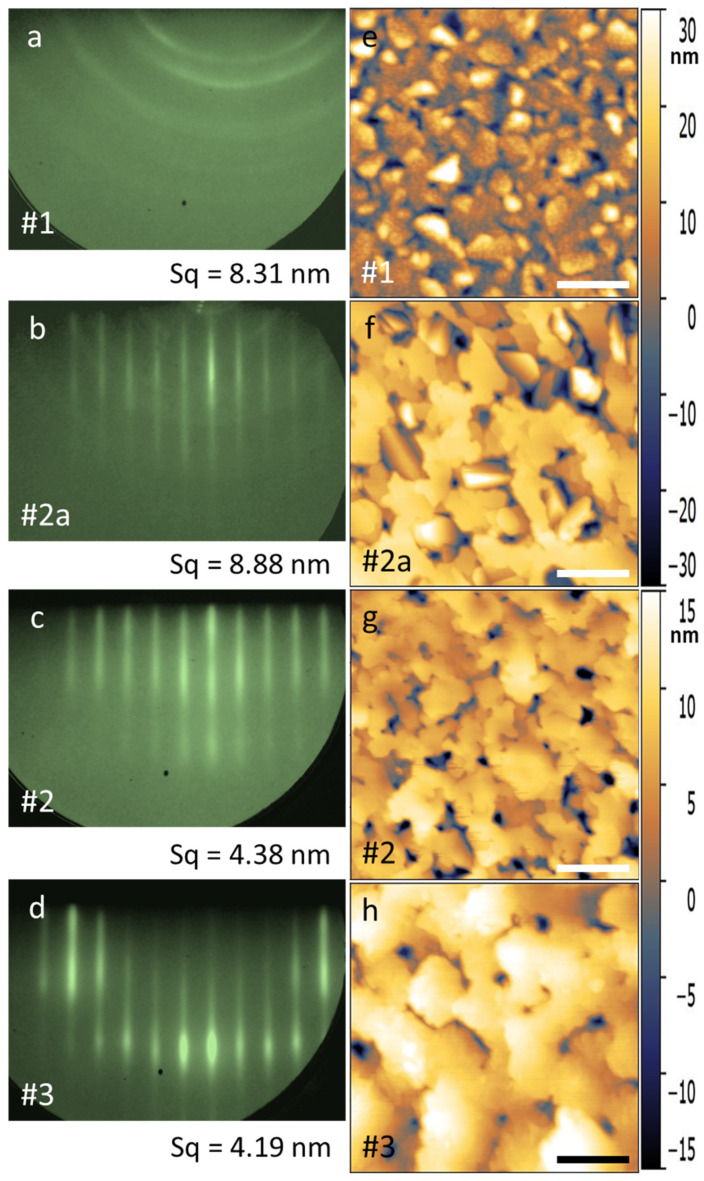
RHEED pattern and AFM images of the Mn_5_Ge_3_ thin films on Si(111). RHEED patterns for the samples #1 (**a**), #2a (**b**), #2 (**c**), #3 (**d**) and AFM images for 2 × 2 μm^2^ area for the samples #1 (**e**), #2a (**f**), #2 (**g**), #3 (**h**) are shown. The thicknesses of Mn_5_Ge_3_ films are #1—45 nm, #2a—60 nm, #2—30 nm and #3—150 nm. The length of the scale bar is 500 nm.

To determine the conditions for the epitaxial growth of Mn_5_Ge_3_ on the Si(111) surface, a series of experiments were performed. Here we show three processes differing in the rate, ratio and time of Mn and Ge deposition during the growth of the buffer layers. These examples demonstrate the effect of buffer layers on the growth processes, structure and morphology of Mn_5_Ge_3_ thin films. Schematic diagram of process flow is shown on Figure 1. Sample #1 was synthesized as a single layer at a fixed deposition rate of Mn V_Mn_ = 0.58 nm/min and Ge V_Ge_ = 0.33 nm/min. During the growth of the first 15 nm, the RHEED pattern transforms from point reflections to streaks and then turns into Debye rings, indicating the formation of a polycrystal (Figure 2a). For sample #2a, one approximately 3 nm thick buffer layer with a low concentration of Mn was grown (V_Mn_ = 0.2 nm/min and V_Ge_ = 0.33 nm/min).

Then, within 10 min, the evaporation rate V_Mn_ gradually increased to the stoichiometric value of 0.58 nm/min. At a film thickness of approximately 19 nm, the diffraction pattern contains mainly only lines from a single crystal. This pattern is observed up to 35 nm, after which the rings begin to appear (Figure 2b). This means that with the help of a highly textured buffer layer used, the Mn_5_Ge_3_ growth can only be stabilized up to a thickness of 35 nm, after which the accumulated stresses in the lattice lead to the formation of crystallites of various orientations. According to this scheme, sample #2 was made with a thickness of about 30 nm. The RHEED pattern for this sample is shown in Figure 2c. To reduce the stresses in the Mn_5_Ge_3_ film, we added three additional buffer layers. For sample #3, after 3 nm at a rate of V_Mn_ = 0.2 nm/min and V_Ge_ = 0.33 nm/min, a 25 nm layer followed with a uniform rate increase up to V_Mn_ = 0.58 nm/min. Further, V_Mn_ was increased to 0.8 nm/min for 10 min, which corresponds approximately to 10 nm. Thereafter, V_Mn_ decreased to 0.4 nm/min and remained unchanged for 4 h 16 min (approximately 150 nm), during which the RHEED pattern remained as the streaks (Figure 2d). In this case, we were able to stabilize the epitaxy-like growth to relatively large thicknesses.

**Figure 3:**
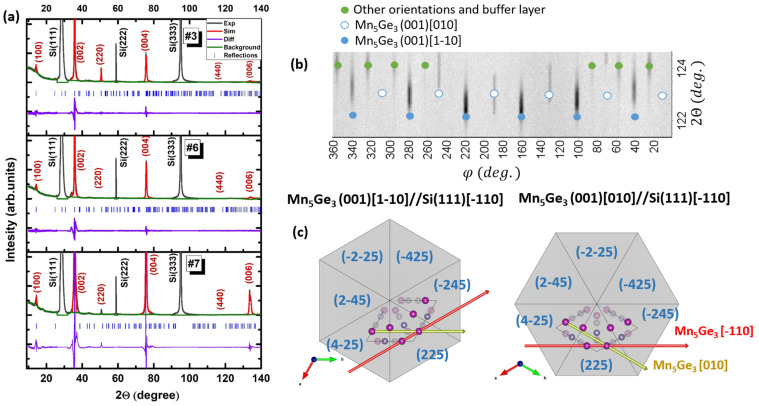
(**a**) XRD profiles for samples #1, #2, #3. (**b**) Phi-scan for sample #3. (**c**) The crystallographic cell of Mn_5_Ge_3_ at a different orientation parallel to Si(111)[-110].

In addition to RHEED, AFM measurements were performed. As seen in Figure 2, RHEED and AFM correlate well with each other, which is expected since both methods are surface-sensitive. AFM demonstrates a visual image of the surface, which clearly shows the difference between polycrystalline samples #1 and #2a and highly ordered samples #2 and #3. Furthermore, we can compare quantitative parameters such as root-mean-square roughness (Sq) taken over the entire area of the scanning frame (Sq values are marked in Figure 2e–h). Comparing Sq for samples #2a and #3, it can be seen that, upon stabilization of the growth conditions for #3, Sq decreases by almost a factor of two. For two highly ordered samples #2 and #3, the crystallite shape and general surface morphology are similar. However, the size of crystallites for sample #3 is much larger than for sample #2, and one can even talk of the formation of terraces. For a deeper analysis of the crystal structure and composition of the film and buffer layers, an XRD study was carried out. Figure 3a demonstrates XRD profiles for samples #1, #2 and #3.

Similar XRD patterns are observed for all samples. The diffraction peaks are in good agreement with those for the Mn_5_Ge_3_ with P6₃/mcm space group crystal. The lattice parameters presented in Table 1 were determined via Pawley refinement with the help of GSAS-II software [19]. Silicon diffraction peaks were used for correction of line shift and afterwards excluded from the refinement procedure. Analysis shows that lattice parameters a (7.112(1) Å) and c (5.027(1) Å) are close to the bulk values. It should be noted that the parameters of sample #3 correspond most accurately to the reference lattice parameters. In addition, the intensity of reflection with index (006) at 133.5 degrees on the X-ray pattern increases from sample to sample and reaches a maximum at #3, which indicates good crystalline quality and high texture on the Mn_5_Ge_3_(001) plane. Consequently, sample #3 was selected for further detailed studies. Figure 3b shows a phi-scan, in which reflections can be interpreted assuming two predominant azimuthal orientations in the (001) plane of Mn_5_Ge_3_ relative to Si(111), as well as a signal from the buffer layer and other differently orientated crystallites of Mn_5_Ge_3_, i.e., (100) texture. The formation of a negligible amount of side orientation is explained by a temperature gradient over 40 × 40 mm substrate. This indicates a high texture on the (001) Mn_5_Ge_3_ plane and only two crystallite orientations. Figure 3c schematically shows the crystal cells of Mn_5_Ge_3_ and marks the two orientation relationships derived from the phi-scan.

**Figure 4:**
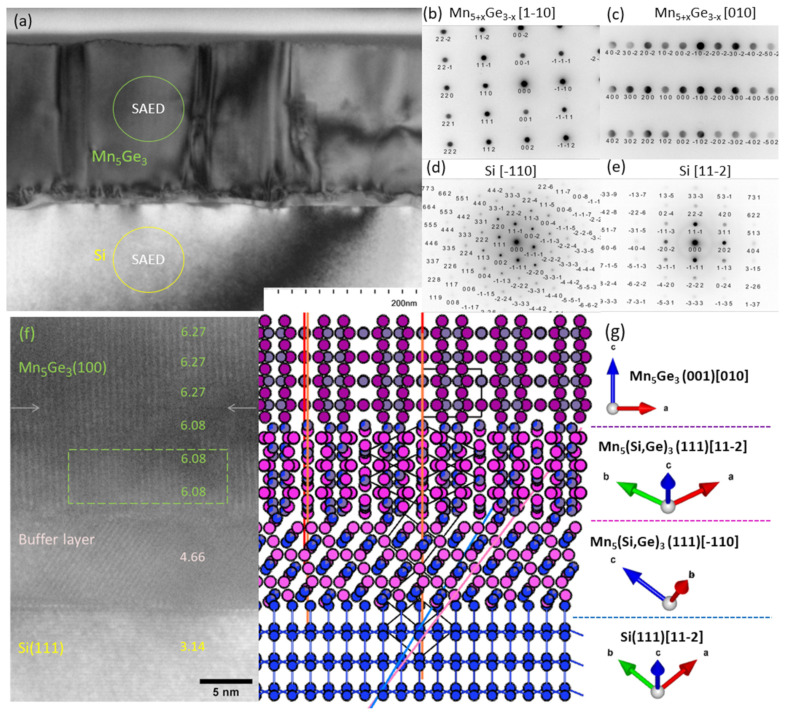
TEM and SAED patterns for sample #3. (**a**) Cross-sectional TEM image of Mn_5_Ge_3_/Si structure. (**b**–**e**) Electron diffraction pattern of Mn_5_Ge_3_ and Si in different crystallographic directions. (**f**) High-resolution TEM near the interface Mn_5_Ge_3_/Si. Numbers are distances between atomic planes in the arb. units extracted from the image by fast Furrier transform analysis. (**g**) Structural model of the Mn_5_Ge_3_ growth with two buffer layers of different orientations and levels of Si substitution of Ge sites.

Sample #3, which has the most complex buffer layer composition and the most ordered structure of the Mn_5_Ge_3_ film, was further investigated using TEM and electron transmission diffraction (SAED) (Figure 4). In Figure 4a, one can see the columnar structure of the film with relatively large crystallites of 100 nm or more. Figure 4b–e show SAED from film and silicon substrate regions. Pairs of images in Figure 4b–e taken in different directions confirm the presence of two different azimuthal orientations corresponding to the epitaxial relations Mn_5_Ge_3_ (001)[1-10]//Si(111)[-110] and Mn_5_Ge_3_ (001)[ 010]//Si(111)[-110]. At higher magnification (Figure 4f), we can clearly distinguish the buffer layer, as well as see the atomic planes of Si, the buffer layer and Mn_5_Ge_3_ film. The distances between the Mn_5_Ge_3_(100) planes increase with the distance from the buffer layer, changing by 3%. Thus, it can be assumed that most (5% of 8%) of the lattice mismatch between Si and Mn_5_Ge_3_ film is eliminated by the buffer layer.

Summing up the RHEED, XRD and TEM data, several candidates can be suggested, including Mn_3_Ge, Mn_5_Ge_2_ and Mn_11_Ge_8_. Taking into account the possible diffusion of silicon from the substrate, Mn_10_(SiGe)_3_ and Mn_5_Si_2_Ge may also be considered. Considering that, in the course of deposition, we tried to reduce the manganese content in the buffer layer, we can assume the formation of Mn_11_Ge_8_ or Mn_5_(Si_,_Ge)_3_ in the buffer layer.

**Figure 5:**
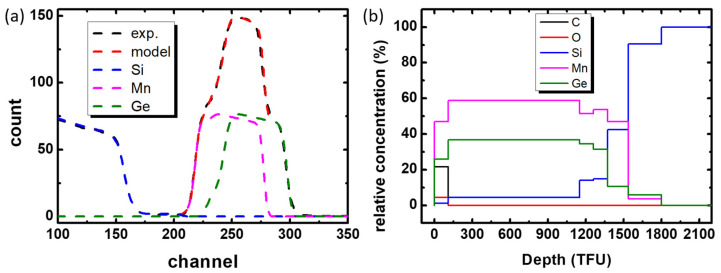
Experimental and model RBS spectra (**a**) and calculated depth profile (**b**) of Mn_5_Ge_3_/Si sample #3.

For a more detailed analysis of the composition of the layers, RBS studies were carried out. Experimental and model RBS spectra are presented in Figure 5a. The sum of simulated individual spectra of Ge, Mn and Si has good agreement with experimental data. The calculated depth profile is shown in Figure 5b. On the surface, we can see some oxidation and carbon contamination. Manganese germanide lies deeper with stoichiometry close to Mn_5_Ge_3_ slightly doped by silicon. Then, the content of manganese and germanium decreases, but the content of silicon increases. This is the transition layer that we are trying to implement in the growing process. However, silicon diffusion additionally occurred in the experiment. It is likely that the area marked with a rectangle in Figure 4f corresponds to a composition of approximately Mn_5_Ge_1.5_Si_1.5_. From here, the decrease in the interplanar distance indicated in Figure 4f becomes clear. Silicon, having a smaller ionic radius than germanium, reduces the volume of the crystal cell. The lowest layer contains up to 90% silicon. Thus, we can conclude that the buffer layer takes on almost all of the silicon diffusing from the substrate, preventing its incorporation into the main Mn_5_Ge_3_ layer.

Considering all structural data and assuming RBS layer modelling, we can propose a double buffer layer model as shown in Figure 4g. This model suggests that all layers have identical crystal structures with P6₃/mcm symmetry similar to Mn_5_Ge_3,_ but orientation and level of Si doping are different. Visibly tilted buffer layer (Figure 4f) has Mn_5_(Si_,_Ge)_3_(111)[-110]||Si(111)[11-2] orientation. Because of the high Si concentration for this layer, the *c* parameter and the interplanar spacing are closer to Mn_5_Si_3_ than to Mn_5_Ge_3_. Next, the intermediate layer is strained Mn_5_(Si,Ge)_3_ with less Si content and Mn_5_(Si_,_Ge)_3_(111)[11-2]||Si(111)[11-2] orientation. The upper film with the biggest volume is relaxed Mn_5_(Ge)_3_ with (001)[010] orientation. Nonetheless, additional azimuthal orientation (001)[1-10] revealed from XRD analysis is presented in the macroscopic film. It should be noted that, estimated from the TEM image (Figure 4f), the angle between the crystal planes of Mn_5_Ge_3_ and Si do not equal 90 degrees. This indicates an additional tilt of the buffer layer. According to our model (Figure 4g), the buffer layer (100) plane may tend to become parallel to the Si(1-31) plane (blue) for better lattice match. In this case, the whole layer will be slightly tilted, and the subsequent layers too. Similar behavior, in particular, two orientations and a non-90 deg. or 0 deg. angle between the *c*-axis and main substrate direction were observed for Mn_5_Ge_3_ thin films grown on Ge(001) [19]. Alvídrez-Lechuga et al. [20] name such growth regime epitaxial mosaic-like.

**Figure 6:**
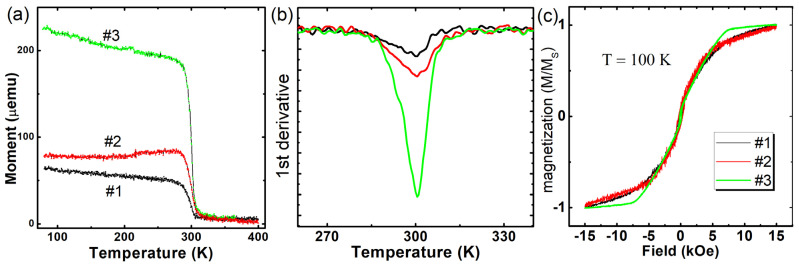
(**a**) Non-normalized magnetization temperature dependencies. (**b**) First derivative of magnetization temperature dependencies. (**c**) Normalized hysteresis loops measured at 100 K for Mn_5_Ge_3_ samples #1, #2 and #3.

To establish the relationship between some physical properties and the structure of the films, the magnetic properties were characterized. From the VSM data, we found that the Curie temperature (T_C_) is about 300 K (Figure 6a). For previously synthesized films on Ge substrates, T_C_ is also about 300 K, which is in good agreement with our samples. For stoichiometric bulk, Mn_5_Ge_3_ T_C_ should be close to 296 K [6]. According to [21], an excess of Mn can lead to an increase in the transition temperature, while doping with Si leads to a decrease in T_C_ [22]. We can, therefore, exclude diffusion of Si from the substrate into the film. According to RBS, a slight excess of Mn and Si doping is possible, which compensates for their effect on T_C_ and leaves T_C_ close to the stoichiometric Mn_5_Ge_3_. It follows that the buffer layer is a good diffusion barrier. Another reason for the deviation of T_C_ may be stresses in the crystal that affect the magnetic properties. Summing up the diffraction data, we can assume that the main reason for the small increase in T_C_ is the excess of manganese Mn_5+x_Ge_3-x_ rather than stresses in the crystal. Epitaxial ordering also does not affect T_C_. On Figure 6b, one can see that the extremum of the derivative for all samples corresponds to approximately 300 K. One should note that the width of the ferromagnetic transition is shrinking with consistently increasing the crystal quality of films #1, #2 and #3. Extracted from hysteresis loops (Figure 6c), saturation magnetization M_S_ is around 1050 emu/cm^3^, which is very close to the bulk quoted value of 1070 emu/cm^3^ [23] and is similar to reported films’ values [15]. Crystal ordering strongly affects magnetic anisotropy, which can be clearly seen from the sharp kink for #3 on Figure 6c.

## 4. Conclusions

The synthesis of Mn_5_Ge_3_ thin films on Si(111) substrates was performed with varying the rate and time of Mn and Ge deposition at the stages of initial growth. Subsequent gradual decrease and increase in the Mn flux and then return to a stoichiometric ratio of 5:3 make it possible to grow a relaxed highly ordered Mn_5_Ge_3_ layer with 100% texture on the (001) Mn_5_Ge_3_ plane. Most (5% out of 8%) of the lattice mismatch between Si and Mn_5_Ge_3_ film is eliminated by the buffer layer. Considering all structural data, we proposed a double buffer layer model suggesting that all layers have an identical crystal structure and symmetry similar to Mn_5_Ge_3_, but orientation and level of Si concentration are different. Analysis of XRD and SAED data shows the presence of two different azimuthal orientations corresponding to the epitaxial relations Mn_5_Ge_3_ (001)[1-10]//Si(111)[-110] and Mn_5_Ge_3_ (001)[010]//Si(111)[-110]. Examination of the magnetic properties has revealed that the Curie temperature, T_C_ is about 300 K, which is close to the bulk value (296 K) and coincides with one of the other reported values for films synthesized on different substrates. Furthermore, the elemental composition of the film probed by RBS allowed us to conclude that the buffer layer takes on almost all the silicon diffusing from the substrate, preventing its incorporation into the main Mn_5_Ge_3_ layer, which is in good agreement with the magnetic properties. We believe that full epitaxial growth can be realized by developing a buffer layer approach and careful substrate temperature control. The observed details of the synthesis of highly ordered Mn_5_Ge_3_ film can be useful for the growth technology of ferromagnetic epitaxial films on Si(111) substrates and can expand the number of applicable materials in silicon spintronics.

## Figures and Tables

**Table 1:** Lattice parameters of Mn_5_Ge_3_ films along with R-factors obtained by Pawley refinement.

Sample	Lattice Parameters		R_wp_, %	R_p_, %
	*a*, Å	*c*, Å		
*#1*	7.213(1)	5.023(1)	13.10	9.14
*#2*	7.178(1)	5.020(1)	13.84	8.40
*#3*	7.112(1)	5.027(1)	12.97	6.15

## Data Availability

Not applicable.
